# RoboEthics in COVID-19: A Case Study in Dentistry

**DOI:** 10.3389/frobt.2021.612740

**Published:** 2021-05-05

**Authors:** Yaser Maddahi, Maryam Kalvandi, Sofya Langman, Nicole Capicotto, Kourosh Zareinia

**Affiliations:** ^1^ Department of Research and Development, Tactile Robotics, Winnipeg, MB, Canada; ^2^ Manitoba Dental Association, Winnipeg, MB, Canada; ^3^ Department of Pathology and Laboratory Medicine, Faculty of Medicine, University of British Columbia, Vancouver, BC, Canada; ^4^ Department of Molecular Oncology, British Columbia Cancer Research Centre, Vancouver, BC, Canada; ^5^ Department of Biomedical Engineering, Faculty of Engineering and Architectural Science, Ryerson University, Toronto, ON, Canada; ^6^ Department of Mechanical and Industrial Engineering, Faculty of Engineering and Architectural Science, Ryerson University, Toronto, ON, Canada

**Keywords:** COVID-19, roboethics, dentistry, education, DenTeach

## Abstract

The COVID-19 pandemic has caused dramatic effects on the healthcare system, businesses, and education. In many countries, businesses were shut down, universities and schools had to cancel in-person classes, and many workers had to work remotely and socially distance in order to prevent the spread of the virus. These measures opened the door for technologies such as robotics and artificial intelligence to play an important role in minimizing the negative effects of such closures. There have been many efforts in the design and development of robotic systems for applications such as disinfection and eldercare. Healthcare education has seen a lot of potential in simulation robots, which offer valuable opportunities for remote learning during the pandemic. However, there are ethical considerations that need to be deliberated in the design and development of such systems. In this paper, we discuss the principles of roboethics and how these can be applied in the new era of COVID-19. We focus on identifying the most relevant ethical principles and apply them to a case study in dentistry education. DenTeach was developed as a portable device that uses sensors and computer simulation to make dental education more efficient. DenTeach makes remote instruction possible by allowing students to learn and practice dental procedures from home. We evaluate DenTeach on the principles of data, common good, and safety, and highlight the importance of roboethics in Canada. The principles identified in this paper can inform researchers and educational institutions considering implementing robots in their curriculum.

## Introduction

The coronavirus disease 2019 (COVID-19) pandemic has caused disturbances in all aspects of everyday life, including healthcare, commerce, manufacturing, and education. In Canada, the response to the COVID-19 pandemic included the shut-down of businesses and the reallocation of human resources to emergency functions (Detsky and Bogoch, 2020). Additionally, many companies asked personnel to work from home if possible. In order to slow down the spread of the SARS-CoV-2 virus, human interactions have to be limited, and people were asked to wear masks, avoid touching surfaces and their faces, and wash hands as often as possible. In a world where human interaction is minimized, robotics and artificial intelligence can be invaluable tools in rebuilding the economy and resuming the “normal” life. Since the start of the COVID-19 pandemic, robots have been used to disinfect hospital rooms (Neustaeter, 2020) and provide comfort to Alzheimer’s patients (Ryan, 2020). Robots can also potentially be used to deliver packages, track inventories, stock shelves and take temperatures. As health agencies are anticipating possible third and successive waves of COVID-19, a wider distribution of robots could alleviate the pressure on essential personnel and help minimize the spread of the virus. When considering a more significant implementation of robots in human life, ethical considerations must be made to ensure that robots are used for the betterment of humanity.

Ever since automatic machines have been invented, writers and directors have been dreaming up possible scenarios for robot technology to develop. Not surprisingly, many such attempts depict the end of the human race and the rise of machines. Discussion of robots in popular culture affects the public’s view of robotics. In fact, over 70% of people say that sci-fi movies have influenced their attitude toward robots (El Mesbahi, 2015). In order to prevent these grim predictions from becoming a reality, and to appease the public’s fears of a possible real-life Terminator scenario, ethical aspects of robotic development have to be considered, and strict rules of conduct are to be established. The relatively new field of roboethics, formally acknowledged since 2002, inspires the design, manufacturing, and use of robots (Veruggio, 2006). Roboethics shares some of its core ideas with information technology ethics, especially concerning the safe use of technology and fair access to technological resources. Robots pose a new challenge from the regulatory standpoint: if a robot commits a crime, who is liable? Would a company that built the robot be at fault when the robot’s actions are unpredictable? Or how should the law tackle establish regulations that describe criminal intent in a machine (Pagallo, 2017; Bösl and Bode, 2018)? Roboethics helps to establish a structured way of dealing with moral dilemmas arising in robotics. If certain laws and moral principles are established for robotics research, that could facilitate the integration of robots in day-to-day life.

## The Laws of RoboEthics

The earliest rules of robotics were described by Isaac Asimov in 1942, when robots were more of a fiction than reality (Asimov, 1942). These Laws, including the Zeroth law added after the first three, are presented below:

1. A robot may not injure a human being under any conditions—and, as a corollary, must not permit a human being to be injured because of inaction on [the robot’s] part.”
2. A robot must follow all orders given by qualified human beings as long as they do not conflict with Rule 1.
3. A robot must protect [its] own existence, as long as that does not conflict with Rules 1 and 2.
4. (Zeroth law) No robot may harm humanity or, through inaction, allow humanity to come to harm.

The above Laws are generic and do not consider the wide spectrum of robotics applications. Since 1942 the field of robotics has evolved, and some of the resulting robots do function in accordance to the Laws. For example, combat robots have become a reality: robots are used to carry loads and other logistical support (Vincent, 2015) and help in bomb disposal (US Department of Homeland Security, 2020). However, it is not difficult to imagine a future in which robots will be able to carry firearms and assist in warfare. The latter would be in clear violation of Asimov’s Laws as the harm to opposing humans would be imminent. On the other hand, the side that fights using robots is able to preserve the lives of its own soldiers. The dichotomy between ethical outcomes in the above situation is further amplified by a robot’s potential inability to comprehend what “existence,” “harm” or “humanity” is. If the robot has no sense of self, how can it act as an ethical agent?

In addition, Asimov’s Laws are robot-centric and do not consider humans who design the robots. Since Asimov, many organizations and agencies have attempted to revise the Laws of Robotics to reflect both the complexity and the developments of robotics. As such, the field of roboethics can be divided into two fields: engineering ethics and machine ethics (Dodig Crnkovic and Çürüklü, 2012). Engineering ethics produce rules and bestow responsibility for robotic creations on engineers and computer scientists. Machine ethics suggests that internal ethical principles and moral decision-making patterns should be designed into robots, making them capable of autonomous ethical decision-making.

Murphy and Woods have proposed a revision for Asimov’s Laws, shifting the attention from machine ethics to human design and responsibility (Murphy and Woods, 2009).

1. Human–robot work systems must comply with rigorous professional and legal standards for safety and ethics. Without such compliance, humans cannot utilize robots in a working system.
2. Robots must respond to humans only so far as determined by each robot’s role.
3. Humans must provide robots with autonomous mechanisms for self-preservation. But those mechanisms must relinquish control as needed to comply with the previous two laws.

The revised Laws are in line with engineering ethics ideas but are still too general to be easily applicable to all robots, or to be written as a part of a government or industry policies. Across the globe, institutions have used the above Laws as a starting point for the development of their own sets of ethical standards for robotics.

## Types of Roboethical Considerations

There are several categories of ethical questions that must be considered before a robot becomes available to the public. These categories are not exclusive, and as the field develops further categories may be added.

### Data

Today, data is becoming one of the most valuable commodities. Robots that have a capacity to record, store, and process data are thus working with a precious resource that must be carefully managed. With data that comes from the robot’s operator, privacy is the topmost concern. A user should be able to consent to their data being recorded, but safety or ownership of that data is not inherent. Would a robot store data locally or on the cloud? Does the data, once recorded, belong to the user or the robotics company? Can that company guarantee the privacy of the data recorded? Is the data safe from possible breaches, and if leaked, can that data be used in harmful ways? If the company uses recorded data to further research or make a profit, should the primary source of data be compensated? If the company was funded through tax-dollars, should the generated data be available to the public? Questions of data ownership, integrity and accessibility should be considered at the design stage to ensure that human rights are not being violated, and the work contributes to the common good.

### Common Good

With robots playing an increasingly larger role in day-to-day life, engineers and computer scientists who design robots will have to consider the broader impact of their work. Many robotics guidelines, such as the ones put up by the Institute of Electrical and Electronics Engineers (IEEE) and European Commission European Group on Ethics in Science and New Technologies, include clauses on the betterment of humanity and human well-being (European Group on Ethics in Science and New Technologies, 2018; The IEEE Global Initiative, 2019). As technologies develop, organizations, countries, and humanity should consider the impact such technologies will have in the future. With billions of dollars spent on military research, is artificial intelligence (AI) arms race something that should be prohibited at early stages? Can robots support human autonomy and prosperity? Would robot-generated benefits be accessible to anyone on the planet? If robots’ expanded functionality makes certain professions obsolete, should there be a contingency plan to retrain workers? One could also ask how “common” is the common good—is contributing to the collective good instead of benefiting one’s own interests something that organizations or countries are capable of?

### Safety

Since Asimov proposed his Laws of Robotics, safety has been one of the main concerns for robotics. Ideally, a robot should never intentionally harm a human, but with the development of autonomous machines such as self-driving cars, the field of robotics has encountered the trolley problem^1^ (Thomson, 1985). In case of an accident, should a car prioritize the life of its own passenger(s), or the bystander(s)? A machine would need to be able to decide in less than a second, and the decision algorithm is to be programmed in by an ethical computer scientist. The trolley problem can be solved in many ways depending on one’s philosophical beliefs, so an overarching directive coming from a governing body would be necessary to unify the responses. In general, there is a need for a regulatory organization that would oversee the development of robotics to ensure human safety, system transparency and correct reporting, adherence to existing laws, and overall regard for humanity’s future.

Robots’ safety should also be considered here—if AI indeed reaches human-like intelligence levels, would it be ethical for humans to use robots in applications where they would be destroyed? Should the public be educated to treat robots with compassion and to prevent vandalism against robots, or should they be treated as utilitarian constructs built to serve as tools?

## Current State of RoboEthics in Canada

There is currently no central agency that oversees roboethics in Canada. Universities and funding organizations have proposed some general guidelines, but don’t have the authority to enforce them. One of the most comprehensive AI ethics manifests comes from scholars at the University of Montreal. They proposed the Montreal Declaration for Responsible Development of Artificial Intelligence in 2018 (The Forum on the Socially Responsible Development of AI, 2018). The main purpose of the Montreal Declaration is to establish an ethical framework for AI development that would benefit everyone in society. As such, the Montreal Declaration is addressed to any person who wishes to develop AI ethically, and to political representatives who may be able to contribute to AI development through lobbying or policymaking. The Montreal Declaration touches upon 10 ethical principles that should be followed when developing AI:

1. Well-being principle
2. Respect for autonomy principle
3. Protection of privacy and intimacy
4. Solidarity principle
5. Democratic participation principle
6. Equity principle
7. Diversity inclusion principle
8. Caution principle
9. Responsibility principle
10. Sustainable development principle

These principles fit under the data, common good, and safety considerations described in the previous section. However, these principles are largely human-centric, and do not account for super-intelligent AI systems.

The University of British Columbia has taken a more inquisitive approach to developing roboethics principles by establishing the N-Reasons Platform (Danielson, 2010). This survey-based platform asks the public to evaluate several types of robots and choose reasons for why they would approve/not approve of their development. Results can be used as an indicator for public perspectives on robotics and inform future policy development. Of note, when the N-Reasons survey was run in 2010, the public was largely supportive of bomb-disposing robots and therapeutic robot animals, but did not agree with the development of fully automated armed aircrafts (Danielson, 2010).

The science of robotics falls under the purvey of the Natural Sciences and Engineering Research Council of Canada (NSERC) funding agency. While there are no robotics-specific policies under their code of ethics, NSERC provides guidelines for ethical standards and values, conflicts of interest, decision-making, private interests, and confidentiality policies. NSERC provides a total funding capital of $1.2 billion dollars to natural science and engineering research, and as a result, has a large influence on determining the direction of innovation in Canada.

While there are no robotics-specific guidelines under the NSERC code of ethics, there are two robotics networks that function under its umbrella. The NSERC Canadian Field Robotics Network (NCFRN) was established to support collaboration between academic researchers, government, and industry partners to create outdoor-capable robotic systems (Natural Sciences and Engineering Research Council of Canada, 2018). Currently, NCFRN develops robots capable of working in land, water, air and human community environments. After NCFRN’s success, the NSERC Canadian Robotics Network (NCRN) has been established (NSERC Canadian Robotics Network (NCRN), 2018). At this time, NCRN has two streams: Interactive Autonomy and Resilient Autonomy. Research in interactive autonomy aims to develop robots that are able to effectively interface and collaborate with humans. Robots developed under the latter category are designed to work in extreme environments for long-term missions. Both NCRN streams of research could contribute to development of robots useful during and after the COVID-19 pandemic.

## Ethical Considerations for Robotics Research and Development During COVID-19

The current economic system favors automation as a tool for faster product manufacturing and distribution. Robots and AI systems are becoming more popular in the service, healthcare, and education systems. As the field of robotics is developing, the resulting robots are becoming faster and more capable. Robotic intellect, usually powered by AI, must be considered when designing long-term roboethics politics or implementing robotic solutions in everyday life. It has been predicted that robots will reach human-like intelligence sometime in the 21st century (Kurzweil, 2005; Hibbard, 2008).

Since the start of the COVID-19 pandemic, robots were used as a solution for minimizing human-to-human contact. After the pandemic is contained, we may find that robots are playing a bigger role in our society than pre-COVID-19. Consequently, the field of robotics may consider revisiting ethical guidelines to ensure that newly deployed robots are benefiting society.

### Bonds Between Humans and Robots

During the pandemic, vulnerable populations of elderly patients found themselves emotionally bonding with robots because they were unable to interact with their loved ones. Some elderly care homes provided Paro, a furry seal-looking robot, to their patients for stress-relief and comfort (Knibbs, 2020; Ryan, 2020). As a result, patients bonded with a robot, expressing feelings of love and excitement to it. We now should consider how ethical it is to let sometimes disoriented patients emotionally invest into a robot. In the past, such trust has sometimes turned unfortunate when robots were disabled by their parent company. Jibo was a robot developed by Jibo Inc.; it was the first intelligent speaker and was capable of learning new patterns as the users interacted with it (Camp, 2019). When Jibo Inc. was sold off and servers hosting source codes disabled, Jibo started glitching and eventually turned off forever. For users who spent months interacting with Jibo, it was a painful and distressing process. If robots are to become an integral part of a patient’s life, its lifespan must be guaranteed. In a case of a company going bankrupt and turning off the servers, there could be a way to make the source code public so that any individual can host the code for their robot. In the culture of planned obsolescence, robots that form relationships or perform critical tasks cannot be allowed to slowly become non-functional for a company’s profit. Guarantee of robot’s function beyond a year-long trial period enforces the trust into robot’s safety. Moreover, when that bond between the robot and a human (e.g., patient or elderly person) is established, companies can use that as a money-making function for their profit.

### Loss of Jobs

Loss of jobs to automation is a hot topic for economists. Automation has played a critical role in the development of the current market: from the first assembly line to shipping, machines have increased productivity across the globe. As robots and AI develop, workers are facing changes in employment opportunities. Currently, it is estimated that in 6 out of 10 occupations, at least 30% of activities are automatable (Manyika et al., 2017). While up to 375 million people globally might need to switch their jobs by 2030, historically, automation has created employment opportunities. Some job sectors, like agriculture and manufacturing, have seen declining employment; however, completely new job positions have been created due to automation. Overall, a country’s labor displacement by automation depends on many factors such as demographics, industry structure, and economic strategy. Due to the COVID-19 pandemic, many businesses were forced to close, and work positions that rely on human-to-human contact were especially affected. For some of those jobs, human employees were replaced with robot employees. Laid-off workers now have a choice of coming back to constricted job market or to go back to school to enter a new job market. For workers who are unable to do either, they are forced to join the gig economy, resulting in reduced wages and social stability. In line with the common good principle, governments should consider the careers of workers whose positions are “automatable,” providing them with post-graduate education opportunities and expanded job markets should come together with higher robot implementation.

## RoboEthics and Education Pre- and Post-COVID-19

Exploration for the use of robotics in education has yielded many promising classroom robots such as Nao, a robot developed by Alderbaran robotics. Nao can be used to teach programming skills, motor skills (such as handwriting) and be a learning peer for autistic students (Shamsuddin et al., 2012; Hamamsy et al., 2019; Sandygulova et al., 2020). As this example suggests, robots can hold different roles in the educational process: they can act solely as tools, but they can also be social. Social educational robots can be further subdivided into teachers, peers, and novices (Belpaeme et al., 2018). Teaching robots are typically used to instill new skills, like teach new vocabulary to an elementary school student. This teaching type of robots has been around the longest. Teaching robots are successfully used as interactive practice tools, and as an alternative knowledge dissemination device. Interestingly, when robots establish an empathetic link with a student, student’s results are observed to be better (Saerbeck et al., 2010). Further on, robots can act as tutors in one-on-one teaching sessions by personalizing their instructional approach to each individual student, thus improving their student’s performance (Leyzberg et al., 2014). The application of tutor-robots in conjunction with online adaptive learning approaches is a promising area of development for dental education in particular (Alwadei et al., 2020). Peer robots are developed to form an empathetic bond with students, learn alongside human subjects, and collaborate with students to solve problems. Novice robots tap into a pedagogical method where a student must explain or teach a topic to a novice. Novice robots are programmed to be taught by students.

When considering the ethics of teaching, the effectiveness of the method is one of the chief concerns. Would a robot-teacher be as good at delivering material when a robot is unable (currently at least) to fully observe non-verbal cues coming from the students? For robot–teachers that have a capacity to automatically evaluate student’s performance, their assessment should pass the following criteria (Kalu et al., 2005):

(1) Be accepted by the experts in the teaching community;
(2) Be reliable, and provide the same result when performed at various times;
(3) Be valid, thus measuring the skill being measured (instead of measuring skill tangential to skill being assessed).

To assess the validity of a particular teaching robot, we should consider how well the robot-delivered assessment reflects the real-life skill, predicts future performance, and compares to the existing gold-standard (Kalu et al., 2005; Holmboe et al., 2010).

Additionally, a big part of in-person school education is the development of social skills, and would a robot-based pedagogical team be able to support the same levels of social and emotional development in students? With COVID-19, schools were forced to shut down and largely transition to distance education. In an online classroom, it is harder for the instructor to observe or support their students. For schools where education revolves around practicums and labs, such as dental colleges, students are facing deferred classes and exams (Wu et al., 2020). Dental students have reported concerns about their clinical care education, but were open to other strategies such as simulation and teledentistry (Hung et al., 2020). Schools can hence adapt by using robots as tools for practicums, and in some cases, as instructors. The future of robot-centric education might be coming sooner than expected. It yet remains to be answered whether robotic educational tools would serve equally as well in training students as human instructors. As robotic tools often rely on the Internet to connect and run their respective programs, students located in areas with poor or nonexistent connections could be at a disadvantage. Additionally, depending on the level of education, students that pay high tuition prices might not be ready to pay for robot-centric education when there is no evidence of efficacy. There is much potential with robotics in education, and the COVID-19 pandemic might be helpful in providing more data and direction to educational robotics field.

## DenTeach for Remote Dental Teaching and Learning

The COVID-19 pandemic has paused both a dental practice and dental education. Because SARS-CoV-2 virus can be found in the saliva of infected patients, dental healthcare professionals are at a higher risk of exposure to the virus (Wyllie et al., 2020). Even after the first wave of the COVID-19 has subsided, dental practices are not able to return to the same patient numbers as pre-COVID, and dental schools are largely conducting instruction online. Dental clinics are able to continue working at reduced capacity until a vaccine is available by increasing preventative measures such as increased handwashing and the use of protective shields. However, dental colleges are not able to conduct any of the practical aspects of the curriculum due to classroom setup and instructors’ capacity. This is a major source of concern for dental students, whose clinical education has been disrupted by the pandemic (Hung et al., 2020). Virtual educational systems and dental simulators have been previously tested in remote education settings and yielded promising results. Students reported positive attitudes toward virtual practice systems and VR-assisted dental simulators. Students also observed a significant increase in their practical skills performance (Liebermann and Erdelt, 2020; Liu et al., 2020; Murbay et al., 2020). However, previously developed dental training simulators are not widely adopted by dentistry schools because of the following concerns: affordability and portability. In addition, currently available simulators are not yet officially recommended for the assessment or training of students (Galibourg et al., 2020). The latter might change soon due to the necessity of remote dental education. To address the concerns of portability, Tactile Robotics has developed a haptic-enabled robotic platform for dental teaching, learning and practicing purposes. This platform, called DenTeach, is portable and affordable, making remote practical dental education a possibility (Maddahi et al., 2020).

For dental educational institutions, DenTeach allows for continuous education through COVID-19, and aids in helping classrooms to become more efficient. DenTeach faithfully captures the environment in which a dental task is performed. It records both sounds, video, and drill vibrations during the procedure demonstrated by the instructor. This allows the student to better understand the task and facilitates the learning process. Additionally, DenTeach is supplied with an articulator that can mimic a patient’s posture, thus training the student to perform the task in the most realistic position. Neither of these functions is widely available in a traditional classroom. As a result, DenTeach can increase teaching efficiency by accelerating skill acquisition by students, and removing limitations based on classroom size.

DenTeach provides extensive quantitative feedback and allows the student to learn and practice at a remote location, such as their home, with a minimal supervision of the instructor. The system uses a combination of sensory responses, and performance data to aid in the learning experience. During the instruction, the students experience how the tool feels during the performance of the task. Understanding proper dental tool handling is also made possible through sensors and actuators on dental tools and by providing an effective augmented reality environment. For the instructors, DenTeach supports an intuitive interface that allows the instructor to demonstrate the model procedure, review students’ work, and provide them with assessments. Dental instructors can teach from a remote location, and the students are able to follow along with the procedure at their own workstation. Additionally, instructors can record a procedure that students can follow along later. This provides an opportunity to change the novice-expert apprenticeship model as an expert would not need to be present for instruction. The recorded material can be reused and could result in cost-benefits to educational institutions. A summary of the capabilities of the platform is listed in Table 1.

**TABLE 1:** Capabilities of DenTeach in both instructor and student workstations.

Instructor	Student
Evaluation of Performance	
Quantifying the students’ skills during the performance of dental tasks using several KPIs^a^	Real-time quantitative evaluation of performing dental tasks using several KPIs
Evaluating students based on the performance index	Compare performance skill with instructor’s KPIs

Instructional Experience	
Measuring kinematic and kinetic characteristics of the dental tool motion	Follow along with a recorded video, audio and haptic feeling
Communicating with students through a custom-made user interface	User-friendly augmented reality
Recording teaching sessions for future use, online education (e-learning) and case rehearsal	Available 24/7
	No supervision required

Physical Setup	
Portable for use everywhere from school to home	

^a^KPIs: Key Performance Indicators.

### DenTeach Platform

**FIGURE 1:**
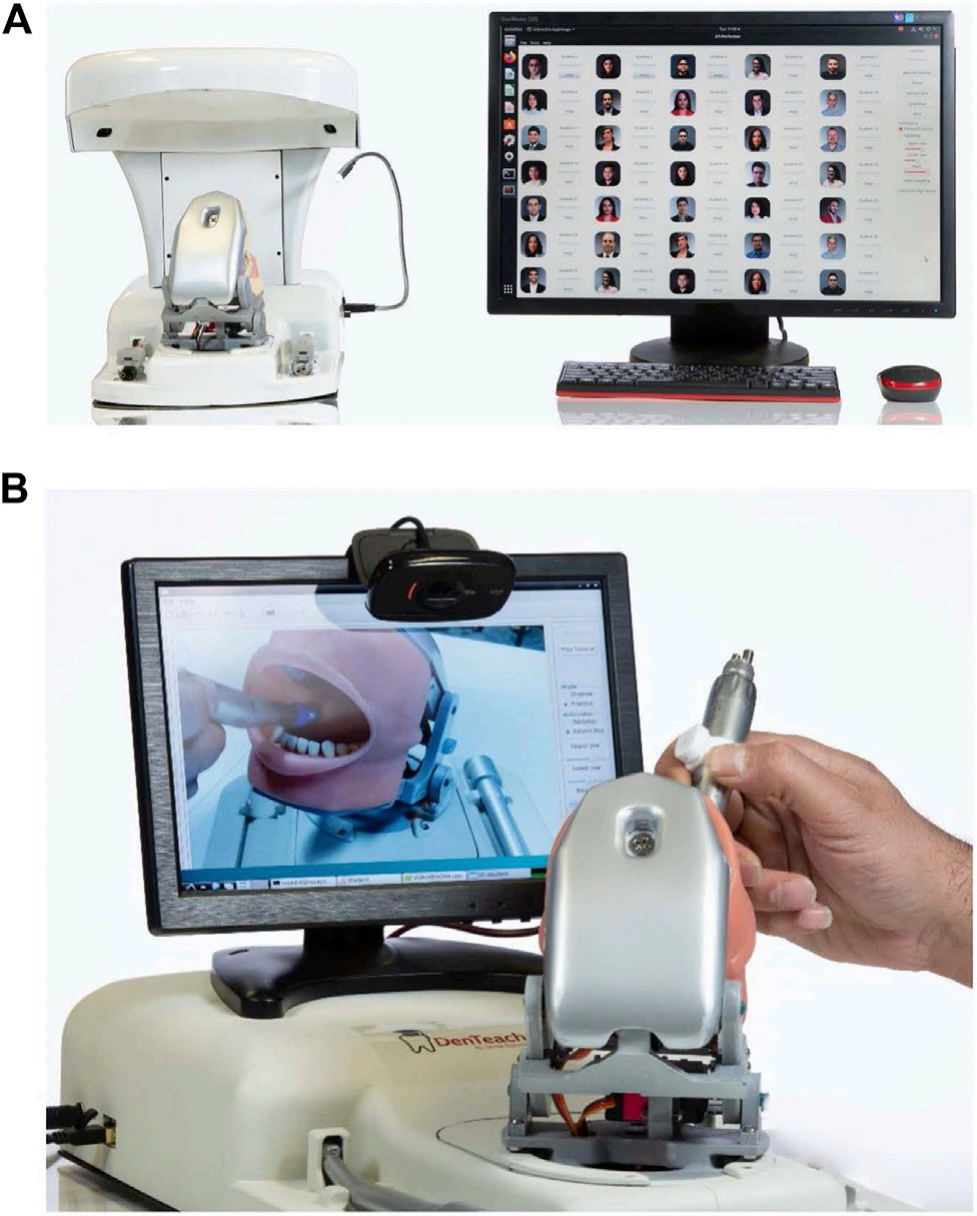
**(A)** Instructor workstation. Instructor workstation comprises of a DT-Rightway dental articulator, a sensory system, 4 cameras covering 360° view coverage, a microphone to speak with students in real-time, a software that shows the performance and information of each student and allows the instructor to monitor them remotely. **(B)** Student workstation. Student workstation consists of the DT-Rightway dental articulator, a sensory system, two sets of RealFeel dental handpiece (slow-speed and high-speed) and a software that captures student’s actions and provides performance metrics.

DenTeach is a vibrotactile dental apparatus that can be situated to work in a wired or wireless setting. There are two types of DenTeach workstations available. The first setup is the instructor workstation that comprises a set of sensory systems attached to a commercially available dental drill. The sensors are able to measure the position, orientation, velocities, accelerations, and jerk (vibrotactile characteristics) of the drill while it is in contact with a physical model of the tooth (Figure 1A). A robotic-based dental articulator is also included in the platform that allows the instructor to set the posture of the oral cavity in different orientations to emulate the patient’s actual posture. The second type of setup is the student workstation. It includes a custom-designed training tool that has a set of sensory systems and a display showing the dental operation performed at the instructor workstation. A similar robotized dental articulator as the instructor helps the student follow the instructor’s technique and to experience working on patient’s posture in addition to the traditional tabletop technique (Figure 1B). The student workstation software quantifies student’s performance and displays it as a set of key performance indices (KPIs) (Cheng et al., 2021). There is a total of 82 KPIs available through the DenTeach system, allowing for detailed analysis of student’s performance. KPIs include metrics such as task completion time, tool handling smoothness and steadiness.

### DenTeach in the Context of Learning Domains

The learning process and educational goals can be widely classified into three domains based on the Bloom’s Taxonomy. These domains are 1) cognitive, 2) psychomotor and 3) affective (Bloom et al., 1956). The cognitive learning domain includes activities that train critical thinking skills, fact recollection, decision making, and general comprehension of the subject matter (Anderson et al., 2001; McHarg and Kay, 2009). The psychomotor domain includes learning technical skills such as procedural knowledge and accompanying adaptive thinking. Training in the psychomotor domain develops one’s reflexes, dexterity and deliberate movement (Simpson, 1971; Anderson et al., 2001; McHarg & Kay, 2009). Educational goals classified under the affective learning domain typically emphasize skills related to emotional intelligence and personal values (Bloom et al., 1956; McHarg and Kay, 2009).

A successful dental school graduate should have the following characteristics after degree completion: technical competence, critical thinking, ethical and professional values, social responsibility, professionalism in the work environment, patient management, and capability for self-assessment (Schneider et al., 2014). Each of these characteristics correlates with certain learning domains identified above. When dental educators were asked to evaluate the types of skills that dental students train during their schooling, the two areas that received the highest rank were knowledge and technical skills (Hoskin et al., 2019). Critical thinking, decision making, and ethics were ranked highly as well under the same questionnaire (Hoskin et al., 2019). DenTeach is primarily intended for the practice of dental procedures, meaning the students would be engaging primarily in the practice of skills that fall under the psychomotor learning domain. When students are completing dental tasks using DenTeach, they are practicing operating the drill in an environment that emulates patient posture. This allows students to develop their motor skills in addition to spatial perception. The DenTeach system assumes that students already have prior theoretical knowledge of procedures, which was potentially acquired during lectures preceding dental tutorials. In the time of COVID-19, as dental education transitioned online, such theoretical knowledge would have been taught through e-learning tools such as Canvas (Iyer et al., 2020). Additionally, dental students have reported using YouTube to watch videos of dental procedures being performed even before the COVID-19 pandemic (Burns et al., 2020). This evidence suggests that the development of online tools can enhance dental education even after in-person education is resumed (Alwadei et al., 2020; Burns et al., 2020; Jeganathan and Fleming, 2020).

### Ethical Aspects of DenTeach

As discussed in the above sections, when attempting to implement a new piece of technology into human life, we must consider the impact said technology will have. As an educational device, DenTeach’s purpose is to benefit the current instruction model in dental colleges by making dental education accessible to students during and after the COVID-19 pandemic. In this section, we evaluate DenTeach on the principles of data, common good, and safety and discuss potential ethical considerations for DenTeach implementation.

#### Data Considerations: Privacy, Security, Longevity

DenTeach collects two kinds of data when in use: camera footage from an instructor’s feed and sensor data from both students and instructor’s workstations. This data, once recorded, would be processed by a data transmission system and is saved on a local server or on the cloud (depending on the institution’s preference). After the data is saved, it is available on-demand for both students and instructors. To protect students’ privacy, each student is only able to see their own performance, but the instructor has access to all student performance data. This system of information storage and protection is common among higher education institutions, but unfortunately, it is also common for universities and colleges to get hacked (Maranga and Nelson, 2019). With a device that is designed to fully rely on servers and cloud data backups, it is critical to highlight the importance of cybersecurity. Additionally, since remote learning instructors will only be able to base their assessments and marking on data provided by DenTeach, it is critical that this data is not tampered with both by external forces, and by students themselves.

One of the big advantages of DenTeach is the possibility of remote software upgrades. These upgrades can be done swiftly and applied during downtime, thus not impeding the education process. Additionally, Tactile Robotics offers a 4-years guarantee and 24/7 support for students who purchase and use DenTeach. Lastly, in order to access the instructor’s data, install updates, and contact DenTeach for support, students will need to have a stable internet connection, which might not always be available. With expansion of services available online and technologies that require the Internet to function, internet access is starting to be viewed as a human right (Human Rights Council, 2016). While the Internet is reasonably ubiquitous in North America, other areas might not have a stable internet connection available, which should be considered by dental schools accepting international students or situated abroad.

#### Common Good: Benefits of DenTeach

Over the years, there have been several attempts to create a simulation dental classroom. Most studies have found that new simulation classrooms did not have a big impact on the way dental pre-clinical procedures are taught (Buchanan, 2001). New generations of technology utilizing internet connection, virtual reality, 3D modeling and next-generation sensors are currently used to develop dental simulation devices that expand beyond what was previously possible. Still, many devices currently being used require supervision and real-life teaching in addition to being stationary and hence not suitable for remote teaching and learning. DenTeach is designed to support educators in the difficult time of COVID-19, but its potential use can extend beyond pandemic response. Professional education institutions, such as dental schools, require program standardization and objective evaluation (Quinn et al., 2003). DenTeach can provide unbiased quantitative feedback to students, and since instructors are able to upload procedures to the server, students are all exposed to the same material in the exact same way. This provides dental schools and education researchers with a unique opportunity to standardize the curriculum and evaluate the simulation classroom with only the experimental variable being the students. Tactile Robotics is currently conducting studies to comprehensively evaluate and compare student performance in DenTeach enabled and traditional classroom settings. The COVID-19 pandemic has challenged the current novice-expert apprenticeship model, and technology could be a useful tool in helping dental educational model evolve.

DenTeach allows students to practice dental procedures at any time, thus allowing for self-study and self-analysis–two important steps in mastering dental techniques. Because DenTeach is designed to be portable, it allows for schools to admit students located around the globe. Additionally, a standardized curriculum could make high-quality dental education available to students located in areas without dental schools, benefiting both the students, and the surrounding population. Further, to make DenTeach available to any dental school interested, Tactile Robotics has a flexible marketing plan for each country or client interested. This supports the principle of equitable access to technology.

Finally, DenTeach offers both right-hand and left-hand setups to enable students to practice in a way that would be most natural and comfortable for them. This emulates field conditions and is inclusive of all students who would be admitted to dental schools.

#### Safety

DenTeach could not reasonably cause harm to human beings–while it does have a dental drill, and weighs a few pounds, it would require a malicious intent from a human, or an unfortunate accident caused by a human for someone to sustain an injury from DenTeach. As such, safety is not a significant concern for DenTeach implementation.

## Conclusion

The COVID-19 pandemic has stimulated the field of robotics to compensate for restrictions implemented to slow the spread of the SARS-Cov-2 virus. Robots were deployed to perform tasks that were too risky for humans, such as comforting at-risk elderly patients and sanitizing hospital spaces. Because of the urgency of the situation, more attention was focused on the rapid development of robots, and less on the ethical aspects of such robotic systems. The principles of data, the common good, and safety are of most relevance for the ethical implementation of robotic systems. This paper is specifically focused on ethical considerations in the design and development of robotic systems for healthcare education. Dental colleges were forced to shut down during the pandemic, and practical courses were delayed until the time it was safe to resume instruction. For students who are enrolled in dental schools during COVID-19 and are continuing to pay tuition fees, it is a dental college’s ethical responsibility to deliver training, even in these challenging times. This created an opportunity for robots to be used to train students in practical dental skills. Previously there have been attempts to create portable dental simulators: both Simodont and IDEA can be used remotely to assist students in learning dental tasks (Luciano et al., 2009; Gal et al., 2011). DenTeach is a new-generation haptic-enabled dental simulator that can be used remotely to train dental tasks completed with a drill. When implementing DenTeach in the educational curriculum, data privacy and equal access should be considered a priority. We explore different aspects of such considerations and provide examples of real-life applications of robotic systems during the COVID-19 pandemic. This may provide some guidelines for engineers and researchers during the research and design process.

## Data Availability

The original contributions presented in the study are included in the article/Supplementary Material, further inquiries can be directed to the corresponding author.

## Footnotes

1 The trolley problem refers to a series of thought experiments where one must decide between sacrificing one person to save several people. In the experiment, one imagines a trolley running on a track that ends with a several people tied up on the tracks, and the user has a choice to pull a lever to divert the trolley to a track with just one person tied up. Either choice will directly cause the death of one or several people respectively (Thomson, 1985).
